# A PBPK Model of Ternary Cyclodextrin Complex of ST-246 Was Built to Achieve a Reasonable IV Infusion Regimen for the Treatment of Human Severe Smallpox

**DOI:** 10.3389/fphar.2022.836356

**Published:** 2022-03-16

**Authors:** Zhiwei Zhang, Shuang Fu, Furun Wang, Chunmiao Yang, Lingchao Wang, Meiyan Yang, Wenpeng Zhang, Wu Zhong, Xiaomei Zhuang

**Affiliations:** State Key Laboratory of Toxicology and Medical Countermeasures, Beijing Institute of Pharmacology and Toxicology, Beijing, China

**Keywords:** smallpox, ST-246, inclusion complex, PBPK model, dosing regimen

## Abstract

ST-246 is an oral drug against pathogenic orthopoxvirus infections. An intravenous formulation is required for some critical patients. A ternary complex of ST-246/meglumine/hydroxypropyl-β-cyclodextrin with well-improved solubility was successfully developed in our institute. The aim of this study was to achieve a reasonable intravenous infusion regimen of this novel formulation by a robust PBPK model based on preclinical pharmacokinetic studies. The pharmacokinetics of ST-246 after intravenous injection at different doses in rats, dogs, and monkeys were conducted to obtain clearances. The clearance of humans was generated by using the allometric scaling approach. Tissue distribution of ST-246 was conducted in rats to obtain tissue partition coefficients (*K*_*p*_). The PBPK model of the rat was first built using *in vivo* clearance and *K*_*p*_ combined with *in vitro* physicochemical properties, unbound fraction, and cyclodextrin effect parameters of ST-246. Then the PBPK model was transferred to a dog and monkey and validated simultaneously. Finally, pharmacokinetic profiles after IV infusion at different dosages utilizing the human PBPK model were compared to the observed oral PK profile of ST-246 at therapeutic dosage (600 mg). The mechanistic PBPK model described the animal PK behaviors of ST-246 *via* intravenous injection and infusion with fold errors within 1.2. It appeared that 6h-IV infusion at 5 mg/kg BID produced similar C_max_ and AUC as oral administration at 600 mg. A PBPK model of ST-246 was built to achieve a reasonable regimen of IV infusion for the treatment of severe smallpox, which will facilitate the clinical translation of this novel formulation.

## 1 Introduction

**FIGURE 1:**
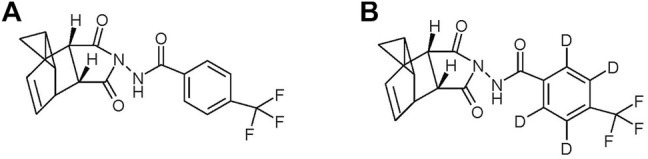
Chemical structure of ST-246 **(A)** and ST-246-D4 (IS) **(B)**.

Although smallpox is no longer a disease found in humans, the possibility still exists that new variants of circulating orthopoxviruses may emerge to cause more frequent diseases in humans (Vorou et al., 2008). In addition, only a very small dose of smallpox is required for infectivity, making it a potent biological weapon (Henderson et al., 1999). Cidofovir has been shown to be effective at controlling poxviral disease in animals but it is toxic and causes kidney damage. Methisazone is only 30–40% effective against smallpox but is toxic and causes nausea and vomiting (Grosenbach et al., 2011). ST-246 (tecovirimat: 4-trifluoromethyl-N-(3,3a,4,4a,5,5a,6,6aoctahydro-1,3-dioxo-4,6-ethenocycloprop[f]isoindol-2(1H)-yl)-benzamide) (chemical structure is presented in Figure 1) is a novel small molecular therapeutic drug that exhibits potent antiviral activity against a broad spectrum of orthopoxviruses (Sbrana et al., 2007; Nalca et al., 2008; Jordan et al., 2010). The oral formulation of ST-246 has been well developed which has enhanced bioavailability in the presence of a high-fat meal (Jordan et al., 2008; Grosenbach et al., 2011). Therefore, ST-246 is recommended to be taken orally after meals. However, intravenous (IV) formulations of ST-246 are still in clinical demand for some critical patients (Vora et al., 2008). For example, when pediatric patients are unable to swallow a pill or little to no food can be taken in some severe cases, accessibility of oral formulation will be greatly compromised. Since ST-246 is readily soluble in organic solvents but is extremely difficult to dissolve in aqueous solutions, the development of an intravenous formulation of ST-246 is rather restricted. Fortunately, a ternary inclusion complex of ST-246 with meglumine (MEG)/hydroxypropyl-β-cyclodextrin (HP-β-CD) was successfully developed in our institute with the dramatic improvement of solubility at 50 mg/mL (Li et al., 2017).

Despite IV formulation development being convenient for IV administration and easy for required dose adjustment, the instantaneous peak concentrations after IV administration and subsequent rapid decrease in plasma concentration would give rise to concerns about the safety and efficacy of ST-246. Optimizing the IV dosing regimen by controlling infusion duration and dose to mimic the plasma exposure obtained after oral administration of conventional therapeutic doses in humans is necessary for the development of this novel ternary cyclodextrin complex of ST-246.

To address this issue, in the current study, the pharmacokinetics of this ternary cyclodextrin complex of ST-246 following IV injection and IV infusion administration in rats, dogs, and monkeys were first investigated. Tissue distribution of the ternary complex of ST-246 was conducted in rats to obtain tissue-to-plasma partition coefficients (*K*_*p*_). Then the traditional allometric scaling approach was utilized to explore the interspecies relationship and to obtain human clearance by extrapolation. Finally, a physiological-based pharmacokinetic (PBPK) model of the ternary cyclodextrin complex of ST-246 after IV injection and IV infusion was established and refined using the middle-out approach. PBPK models are one such system of PK models that can not only be used to understand the whole-body PK of drugs but also be used to accomplish the successful preclinical-to-clinical translation of drug PK (Grimstein et al., 2019; Yellepeddi et al., 2021). The increasing number of these applications using PBPK modeling reveals the high level of confidence that the sponsors and regulatory agencies have in this model-informed drug development approach. After validation, pharmacokinetic profiles after IV infusion at different dosages by the human PBPK model were used to capture the observed plasma exposure of ST-246 at therapeutic dosage (600 mg) after oral administration.

## 2 Materials and Methods

### 2.1 Chemical and Reagents

ST-246 (MW 376.33 g/mol) was synthesized by the chemical synthesis laboratory of the Beijing Institute of Pharmacology and Toxicology (Beijing, China) with a purity greater than 99%, and the identity was confirmed by infrared (IR) spectrum, mass spectrum (MS), elemental analysis, and nuclear magnetic resonance (NMR). Stable isotope-labeled ST-246-D4 (MW 380.36 g/mol) as an internal standard (IS) was synthesized by Shimadzu (Shanghai) Global Laboratory Consumables Co., Ltd. (Shanghai, China) with a purity greater than 99%. Other reagents were of HPLC grade or better.

### 2.2 Animals

Male Sprague–Dawley (SD) rats (200–220 g) were obtained from Beijing Vital River Laboratory Animal Technology Co., Ltd. (Beijing, China). Male dogs (9–11 Kg) were purchased from SPF Biotechnology Co., Ltd. (Beijing, China). Male cynomolgus monkeys (3–5 Kg) were purchased from Guangxi Guidong NHP Experimental Co., Ltd. (Guangxi, China). Animals were housed in a temperature- and humidity-controlled room with a 12-h light/dark cycle. The animal experiments were conducted in the Beijing Center for Drug Safety Evaluation and according to a protocol approved by the Institutional Animal Care and Use Committee of the center, which followed the guidelines of the Association for Assessment and Accreditation of Laboratory Animal Care (AAALAC) International. The protocols for these studies were reviewed and approved before each study.

### 2.3 Preclinical Pharmacokinetic Study

#### 2.3.1 Rat Pharmacokinetics After IV Injection and Infusion

In total, 24 male rats were randomly divided into four groups with six rats for each group. Three groups of rats were given intravenous bolus injection of ST-246 solution *via* the tail vein at the doses of 2, 5, and 20 mg/kg. Blood samples were collected at 2, 5, 15, 30, and 45 min and 1-, 1.5-, 2-, 3-, and 4-h post-dose. Another group of rats was infused with ST-246 solution *via* the tail vein at doses of 5 mg/kg over a 60-min period. Blood samples were collected at 5, 18, 60 (end of infusion), 65, and 78 min and 2-, 4-, and 6-h post-dose. Blood samples for each time point were collected into heparinized polypropylene tubes. The plasma samples were collected after centrifugation of the blood samples at 2000 × g for 10 min and stored at −20°C until analysis.

#### 2.3.2 Tissue Distribution of Rats After IV Injection

In total, 24 rats (six animals for each time point) were given intravenous bolus injection of ST-246 solution *via* the tail vein at the doses of 5 mg/kg. The animals were anesthetized and exsanguinated at 5 and 30 min and 2- and 6-h post-dose. The heart, liver, lung, spleen, kidney, muscle, fat, brain, stomach, and intestine were collected. Harvested tissue samples were weighed and stored at −20°C until homogenization.

#### 2.3.3 Dog Pharmacokinetics After IV Injection

Six male dogs were used for pharmacokinetics study *via* IV injection with a 6-day washout. ST-246 solution was injected at 1, 2.5, and 10 mg/kg *via* the cephalic vein. Blood samples were collected at 2, 5, 15, and 30 min and 1-, 2-, 4-, 6-, 8-, and 12-h post-dose. Plasma samples were collected as the same method as mentioned before.

#### 2.3.4 Monkey Pharmacokinetics After IV Injection and Infusion

A total of three male and three female cynomolguses were used in the monkey pharmacokinetics of IV injection and infusion with a 10-day washout period. ST-246 solution was injected at 3 mg/kg *via* a cephalic vein. Blood samples were collected at 2, 5, and 15, 30 min and 1-, 2-, 4-, 5-, 6-, 8-, 12-, and 24-h post-dose. In an effort to minimize discomfort during the infusions, the cynomolguses had surgery to install vascular access ports (VAP) and were acclimated to jackets that held the test article. In this way, the IV infusion was conducted without the need to restrain the monkeys during the infusion. Monkeys were infused with ST-246 solution *via* the cephalic vein at the dose of 3 mg/kg over a 4-h period. Blood samples were collected at 0.5-, 1-, 2-, 4- (end of infusion), 4.25-, 4.5-, 5-, 6-, 8-, 12-, 24-, and 48-h post-dose. Plasma samples were collected as the same method as mentioned before.

### 2.4 Fraction Unbound in the Plasma of Different Species

The fraction of protein binding of ST-246 to plasma of rats, dogs, monkeys, and humans was determined using a 48-well Rapid Equilibrium Dialysis device (Thermo Bioscience, Woburn, MA). Plasma was spiked with ST-246 at 5 μM as the final concentration. Aliquots of 200 μL of the spiked plasma were loaded into the donor side of a dialysis apparatus, and 400 μL of buffer was added to the receiving side. The apparatus was incubated in a shaking incubator at 37°C for 5 h. After incubation, the samples from donor and receiving chambers were diluted with the same matrixes in the other chamber. The resulting samples were precipitated in acetonitrile with IS. The ST-246 concentrations were determined by the same method as described in the bioanalytical section.

### 2.5 Bioanalytical Method

ST-246 concentrations in rat, dog, and monkey plasma were determined using the liquid chromatography–tandem mass spectrometry (LC-MS/MS) method. A total of 125 µL acetonitrile containing IS (20 ng/mL) was added into 25 µL plasma to precipitate plasma protein. After high-speed centrifugation, 20 µL of supernatant was added to 180 µL of 50% acetonitrile solution and injected onto LC-MS/MS. The chromatographic separation was performed using a C18 column (3.0 mm × 50 mm, 2.6 µm, Phenomenex) with a security guard column. The mobile phase consisted of water containing 0.1% formic acid (A) and acetonitrile containing 0.1% formic acid (B). Separation was achieved with a 3-min run time with the following gradient program: initial conditions start with an increase from 30 to 90% B over 1.2 min, hold at 90% B for 0.5 min, return to 30% B over 0.1 min, and hold at 30% B for 1.2 min. The constant flow rate is 0.6 mL/min. An AB Sciex API 5000 Triple quadrupole mass spectrometer was tuned to the multiple reaction monitoring (MRM) mode to monitor the m/z transitions, 377/173 for ST-246 and 381/177 for the IS, in the positive ion mode.

### 2.6 Data Analysis

Pharmacokinetic parameters were analyzed by using the non-compartmental method using WinNonlin 7.0 (Pharsight, CA). The following parameters were calculated: terminal elimination half-life (t_1/2_ = ln (2)/l_z_), where l_z_ is the first-order rate constant achieved from the terminal (log-linear) portion of the pharmacokinetic (PK) curve; initial plasma concentration post-IV (C_0-IV_) extrapolated from the first three points of the logarithmic plasma concentration; the area under the curve (AUC_last_ = area under the curve from the time of dosing to the last measurable concentration) of plasma (AUC_plasma_) and tissues (AUC_tissue_) calculated by using the linear trapezoidal method; steady-state volume of distribution (*V*_*ss*_ = Amount in body/Concentration at steady state); and the clearance (*CL* = Dose/AUC).

The partition coefficient (*K*_p_) of rats for the measured tissues was obtained from the ratio of AUC_tissue_ toward AUC_plasma_. *K*_*p*_ in other species are transformed from rat *K*_*p*_ according to *K*_*p*uu of human (dog, monkey)_ = *K*_*p*uu of rat_, under the assumption that *f*_*ut*_ is identical between species and rat, namely, human (dog and monkey): *K*_*p*_ = rat *K*_*p*_ × (rat C_plasma, u_/human (dog, monkey) C_plasma, u_).

The unbound fractions in rat, dog, monkey, and human plasma were calculated as the ratio of buffer-to-plasma concentrations of ST-246 (*f*_u_ = ST-246 concentration in buffer/ST-246 concentration in plasma).

### 2.7 Allometric Scaling Method to Predict Human Clearance

When results showed that plasma clearance tends to be proportional to body weight, linear regression of clearance of different species would be performed, and human clearance was scaled by the exponential function of body weight. Based on the simple allometric approach, obtained clearances from animal PK experiments were plotted against the respective body weight on a log-log scale based on the power-based simple allometric equation of *CL* = α × BW^A^ (BW: body weight). A linear regression analysis was then performed according to the logarithmic transformations of the simple allometric equations. Extrapolation to human clearance was based on a 70 kg body weight.

### 2.8 Animal PBPK Model Construction and Validation

A whole PBPK model was developed to describe the kinetics of ST-246 in plasma using the middle-out approach (Tsamandouras et al., 2013; Pepin et al., 2021) by a GastroPlus^™^ simulator (version 9.8). This PBPK model integrated 14 organs (brain, adipose, spleen, heart, lung, muscle, kidney, liver, gut, skin, reproductive organ, red marrow, yellow marrow, and the rest of the body) which are connected with arteries and veins exchanging through blood perfusion. All organs were considered kinetically equivalent to well-stirred compartments. Corresponding physiological parameters were set for specific animals. Key parameters (i.e., physicochemical parameters, R_*bp*_, and f_u,p_), *in vivo* clearance, and measured *K*_*p*_ values of tissues to achieve the volume of distribution as input information are presented in Tables 1–3. *K*_*p*_ values of other animals were attempted to be corrected by an unbound fraction in plasma or directly used *K*_*p*_ values of rats. Then by varying the doses and animals (IV bolus or IV infusions), the plasma concentration versus time profiles of ST-246 in intact animals were simulated, using the PBPK model, for 2, 5, and 20 mg/kg IV bolus and 5 mg/kg 1-h IV infusion in the rat PK study; 1, 2.5, and 10 mg/kg IV bolus dosing in the dog PK study; and for 3 mg/kg IV bolus and 3 mg/kg 4-h IV infusion in the cynomolgus monkey PK study. Observed data were overlaid to and graphically compared with simulated results.

**TABLE 1:** Comparison of pharmacokinetic parameters for ST-246 after IV administration to rats, dogs, and monkeys and IV infusion in rats and monkeys (*n* = 6).

Species	Route	Dose (mg/kg)	t_1/2_ (h)	C_0-IV_, C_max-infusion_ (μg/mL)	T_max_ (h)	AUC_(0-t)_ (h·μg/mL)	AUC_(0-inf)_ (h·μg/mL)	*V*_*ss*_ (L/kg)	*CL* (L/h/kg)
Rat	IV	2	0.56 ± 0.05	9.29 ± 2.06	0	2.95 ± 0.42	2.98 ± 0.43	0.42 ± 0.05	0.68 ± 0.10
	IV	5	0.58 ± 0.06	15.52 ± 2.18	0	6.61 ± 0.79	6.68 ± 0.83	0.50 ± 0.03	0.76 ± 0.10
	IV	20	0.71 ± 0.09	71.18 ± 7.13	0	33.22 ± 11.61	33.35 ± 11.67	0.47 ± 0.04	0.65 ± 0.17
	1-h IV infusion	5	0.67 ± 0.05	4.12 ± 1.17	1	6.49 ± 1.35	6.52 ± 1.36	0.76 ± 0.24	0.79 ± 0.15
Dog	IV	1	1.72 ± 0.41	2.29 ± 0.86	0	1.77 ± 0.29	1.84 ± 0.31	0.99 ± 0.16	0.56 ± 0.10
	IV	2.5	2.36 ± 0.52	7.07 ± 1.14	0	4.77 ± 0.66	4.89 ± 0.71	1.13 ± 0.20	0.52 ± 0.08
	IV	10	2.03 ± 0.27	28.96 ± 5.99	0	16.51 ± 3.62	16.78 ± 3.78	1.80 ± 0.38	0.62 ± 0.14
Monkey	IV	3	5.46 ± 2.89	2.96 ± 1.70	0	4.82 ± 0.40	4.98 ± 0.39	1.92 ± 0.47	0.61 ± 0.05
	4-h IV infusion	3	3.03 ± 1.43	1.20 ± 0.38	4	5.06 ± 1.53	5.15 ± 1.52	1.73 ± 0.62	0.63 ± 0.18

**TABLE 2:** Tissue partitions (*K*_p_) of ST-246 measured in rats and the extrapolated values in dog, monkey, and human PBPK modeling.

Tissue	Measured *K*_*p*_ of rat	Transferred *K*_*p*_ of dog^a^	Transferred *K*_*p*_ of monkey^a^	Transferred *K*_*p*_ of human^a^	*K*_*p*_ of rat for dog	*K*_*p*_ of rat for monkey	*K*_*p*_ of rat for human
Brain	0.13 ± 0.02	0.04	0.03	0.10	0.13	0.13	0.13
Adipose	1.39 ± 0.43	0.41	0.31	1.06	1.39	1.39	1.39
Spleen	0.18 ± 0.04	0.05	0.04	0.14	0.18	0.18	0.18
Heart	0.40 ± 0.08	0.12	0.09	0.30	0.4	0.4	0.4
Lung	0.56 ± 0.14	0.16	0.13	0.43	0.56	0.56	0.56
Muscle	0.18 ± 0.03	0.05	0.04	0.14	0.18	0.18	0.18
Kidney	0.23 ± 0.05	0.07	0.05	0.17	0.23	0.23	0.23
Liver	0.15 ± 0.11	0.04	0.03	0.11	0.15	0.15	0.15
Intestine	0.16 ± 0.02	0.05	0.04	0.12	0.16	0.16	0.16
Skin	0.18	0.05	0.04	0.14	0.18	0.18	0.18
Reproductive organ	0.56	0.16	0.13	0.43	0.56	0.56	0.56
Marrow	0.56	0.16	0.13	0.43	0.56	0.56	0.56
Rest of the body	0.56	0.16	0.13	0.43	0.56	0.56	0.56
Generated *V*_*ss*(L)_	0.101	3.404	1.295	123.327	10.266	5.25	161.257

a *K*_*p*_ in other species are transferred from rat *K*_*p*_ according to *K*_*p*uu of human (dog, monkey)_ = *K*_*p*uu of rat,_ under the assumption that *f*_*ut*_ is identical between rat and species, namely, human (dog, monkey) *K*_*p*_ = rat *K*_*p*_ × (rat C_plasma, u_/human (dog, monkey) C_plasma, u_). *f*_*u,plasma*_ values of ST-246 in rats, dogs, monkeys, and humans measured in the present study were 98.1 ± 0.20, 93.5 ± 0.10, 91.5 ± 0.56, and 97.5 ± 0.13%.

**TABLE 3:** Physicochemical and ADME parameters of ST-246 used for PBPK model.

Parameter	Value	Method
MW	376	
logP	2.44	Predicted^a^
pKa	8.52	Predicted^a^
R_*bp*_	0.63 for rats, monkeys, and humans; 0.7 for dogs	Measured
*f*_*up*_ (%)	1.9, 6.5, 8.5, and 2.5 for rats, dogs, monkeys, and humans	Measured
*CL* (L/h)	0.147, 4.707, and 1.679 for rats, dogs, and monkeys; 29 for humans	Measured; predicted^b^
Cyclodextrin effect (*K*_*c*_, L/mol)	7.105	Measured (Li et al., 2017)

a predicted values were obtained from ADMET Predictor 10.0.

b predicted value were obtained from the allometric scaling method.

### 2.9 Human PBPK Model Construction and PK Simulation

The structure of the animal PBPK model was directly transferred into the human PBPK model of a Chinese man (70 kg body weight, 30-year-old, and 23.98 body mass index (BMI)) (Wang et al., 2021), where 70 kg BW is used to conduct clearance allometric scaling for humans. The clearance of ST-246 in humans was generated from the allometric scaling approach based on the *in vivo* clearances of rats, dogs, and monkeys. Based on the validation of the dog and monkey PBPK modeling, it was found that the predicted volume of distribution by applying the unbound fraction corrected *K*_*p*_ values deviated significantly from the observed values, while the predictive power of this PBPK model was significantly improved by directly applying the measured *K*_*p*_ values in rats. Thus, the volume of distribution in humans was generated from the same *K*_*p*_ values of tissues as rats align with human tissue physiological parameters (Table 2). Finally, a series of plasma concentration–time profiles in humans were simulated following intravenous bolus, 6h-IV infusion QD, and 6h-IV infusion BID over a dose range of 1–5 mg/kg. Observed PK profile of ST-246 at therapeutic dosage (600 mg) after oral administration was taken to compare with these simulated PK profiles. An optimal dosing schedule in the first-in-human study was suggested based on the simulation. Pop PBPK simulations were ultimately conducted to estimate ST-246 exposure in the Chinese adult population (100 virtual subjects, 18–50 years old, 55–85 kg, 17.727–27.397 kg/m^2^ BMI, 50/50 male and female) following the obtained dosing regimen.

## 3 Results

### 3.1 LC-MS/MS Methodology

The quantification of ST-246 in animal plasma was fully validated including selectivity, precision, accuracy, recovery, matrix effect, and stability. The calibration curves of ST-246 in animal plasma ranged in the concentrations of 20–20,000 ng/mL. The intra-day and inter-day accuracy and precision at quality control (QC) concentrations (20, 50, 1,000, and 18,000 ng/mL) were within 86.6–112.4 and 11.72%, respectively. The matrix effect and extract recovery at QC concentrations (50, 1,000, and 18,000 ng/mL) were within 9.8% and 90.7–95.4%, respectively. Stabilities testified at ambient temperature after 1 h, three cycles of freezing and thawing, and being stored at −20°C for 3 weeks were within 87.8–104.4%. The constructed method met the bioanalytical validation standard established by the FDA (Kaza et al., 2019).

### 3.2 Pharmacokinetic Behaviors of ST-246 in Rats, Dogs, and Monkeys Post-Intravenous Administration

**FIGURE 2:**
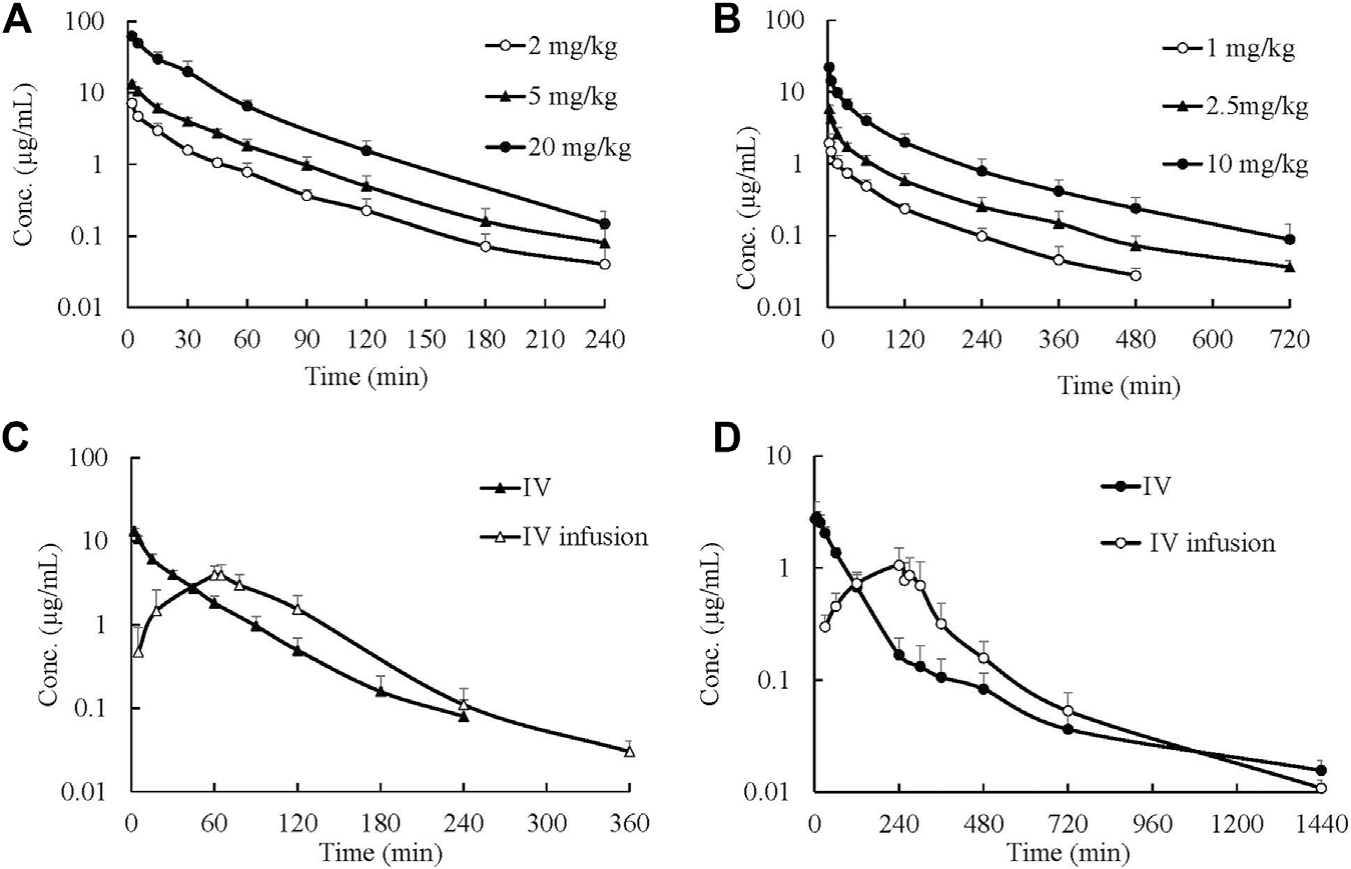
Plasma ST-246 concentration versus time curves in rats, dogs, and monkeys after intravenous bolus (IV) at different dosages and intravenous infusion administration in rats and monkeys (*n* = 6, mean ± SD). **(A)** PK curves of ST-246 *via* IV in rats; **(B)** PK curves of ST-246 *via* IV in beagle dogs; **(C)** PK curves of ST-246 *via* IV and 1 h IV infusion at a dose of 5 mg/kg in rats; **(D)** PK curves of ST-246 *via* IV and 4 h IV infusion at a dose of 3 mg/kg in monkeys.

**FIGURE 3:**
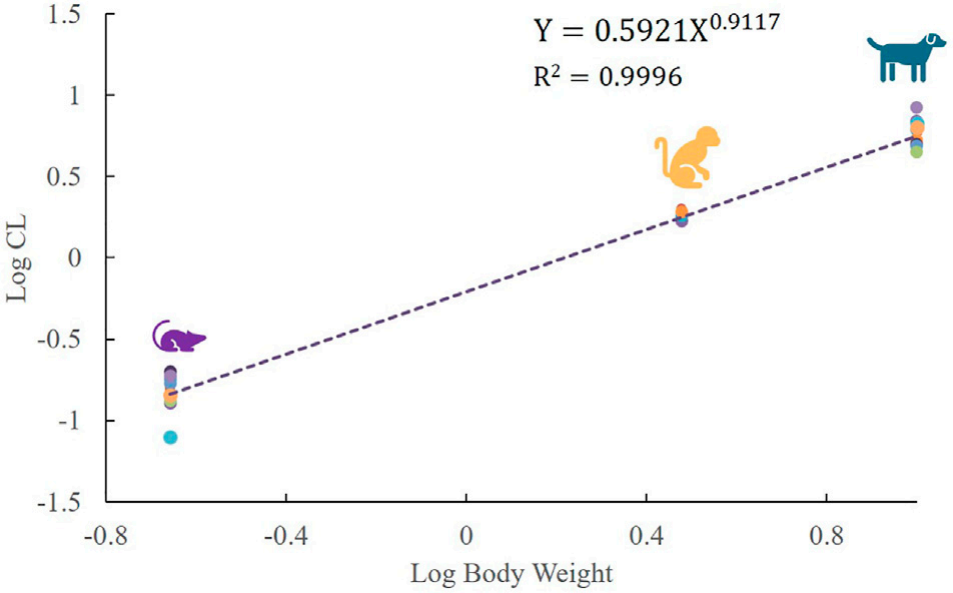
Linear regression analysis of log-transformed plasma clearance for SD rats, beagle dogs, and cynomolgus monkeys versus log-transformed corresponding animal body weight, following IV administration of ST-246 at respective doses (each point represents the individual clearance of each animal, *n* = 18 for rats and dogs, *n* = 6 for monkeys).

Mean plasma concentration–time profiles and major PK parameters of ST-246 in rats, dogs, and monkeys after various IV doses and IV infusion are presented in Figure 2; Table 1. Linear pharmacokinetics were observed both in rats and dogs post-IV administration of various dosages of ST-246 with achieved dose-related C_0_, AUC, and relatively consistent clearance. The elimination half-lives were similar for the IV infusion and IV injection over different doses for individual animals. For IV infusions, the C_max_ appeared at the end of the infusion. Compared to IV bolus at the same dose level, IV infusion in rats and monkeys yielded similar AUC and ∼3-fold lower maximum plasma concentration. The allometric relationship between body weight (BW) and *in vivo* clearance (CL) of rats, dogs, and monkeys are shown in Figure 3. Good correlation was observed according to the coefficients of determination (R-squared of 0.9996), and the resulting allometric equation ( $CL=0.5921\times BW^{0.9117}$ ) was used to predict the CL of human. The estimated plasma CL for a 70-kg human was 29 L/h.

### 3.3 Tissue Partition of ST-246 in Rats and Its Conversion Into Other Species

Tissue distribution conducted in rats showed ST-246 could be detected in various tissues with the time period examined and exhibited rapid distribution and elimination which is consistent with the previous observation that ST-246 could broadly distribute to all organs, including the brain (Grosenbach et al., 2011). Obtained *K*_*p*_ values of all the tissues in rats are presented in Table 2. Apart from adipose, *K*_*p*_ values of other tissues are all less than 1, indicating the tissue partition of ST-246 is limited, which is consistent with the results of PK in rats (*V*_*ss*_ of 0.40 L/kg in Table 1). Based on the well-accepted perspective that *K*_*p,uu*_ of rodents can be used as a surrogate to other species (Nigade et al., 2019), *K*_*p*_ values of all the tissues in dogs, monkeys, and humans can be converted based on the correction of *f*_*u*_ *,*_*plasma*_ in different species (Table 2). In the current investigation, plasma protein binding for the rats, dogs, monkeys, and humans was simultaneously determined with the resulting similar protein binding values of 98.1 ± 0.20, 93.5 ± 0.10, 91.5 ± 0.56, and 97.5 ± 0.13%, respectively. However, the corresponding unbound fractions displayed obvious differences from the lowest of 0.019 to the highest of 0.085, with a maximum difference of more than four times. We first used the converted *K*_*p*_ to generate *V*_*ss*_ for dogs and monkeys and found that it deviated too much from the measured values, while the *V*_*ss*_ calculated directly from rat *K*_*p*_ values was much closer to the measured ones (Table 2). Finally, integration of the same *K*_*p*_ values of major tissues as rats in to the PBPK model to generate volume distribution (*V*_*ss*_) for different species.

### 3.4 PBPK Model Construction in Rats

**FIGURE 4:**
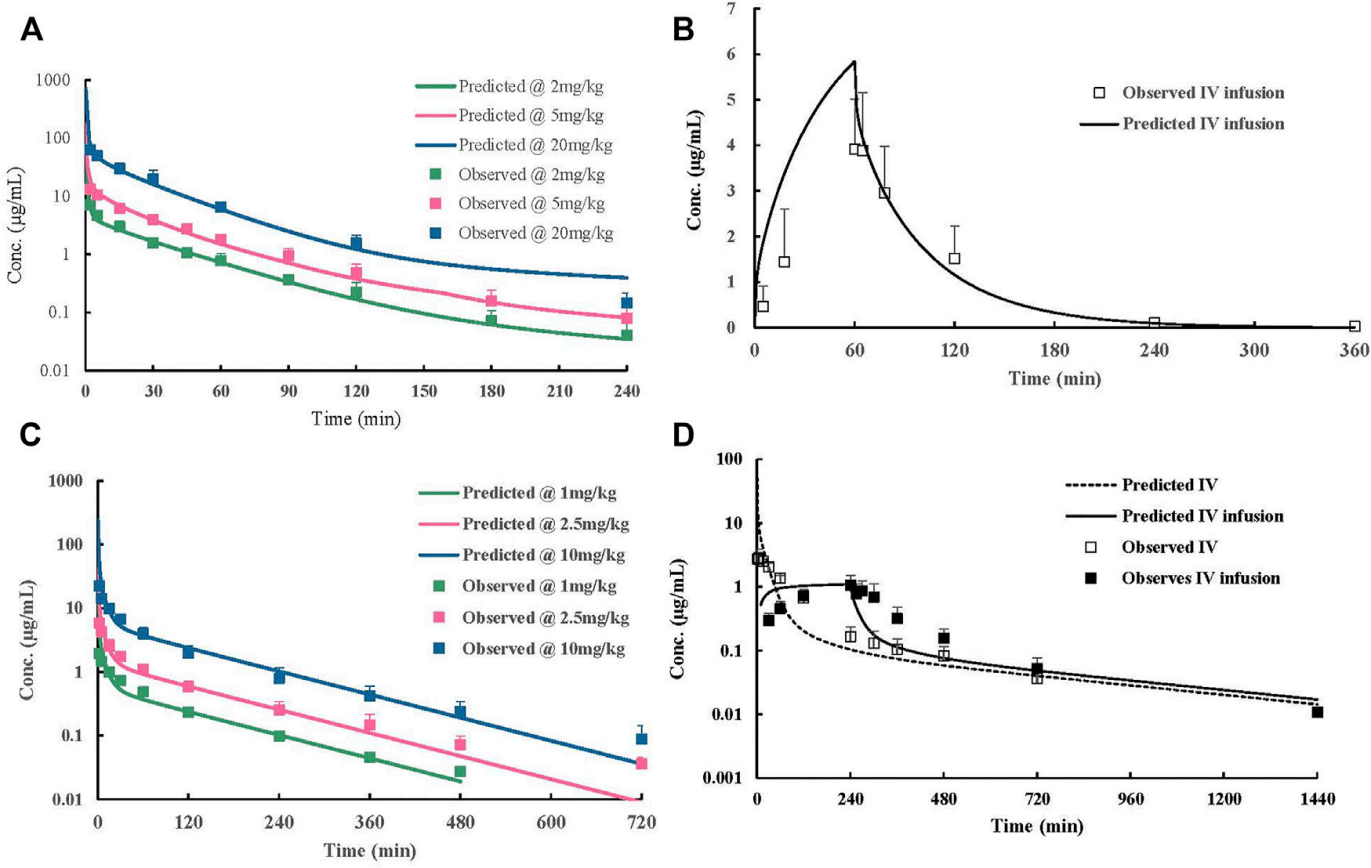
Observed and predicted concentration–time profiles of ST-246 doses in animals. **(A)** Rats post-IV 2–20 mg/kg; **(B)** rats 5 mg/kg 1 h post-IV infusion; **(C)** dogs 1–10 mg/kg post-IV; **(D)** monkeys 3 mg/kg 4 h post-IV infusion. Scatter of points represent measured values; lines represent predicted the PK curve.

The whole PBPK model was first constructed in rats using psychochemical parameters, *in vivo* clearance, and volume of distribution generated from determined *K*_*p*_ values of major tissues coupled with fitted *K*_*p*_ values of other organs. Simulated plasma concentration–time profiles agreed well with obtained concentration values over different doses after IV administration in rats (Figure 4A). The predicted plasma concentrations described by AUC_0-inf_ were all very close to those observed values (Table 4). For the group of 1-h IV infusion in rats at 5 mg/kg, the predicted PK profile captured well with the observed result (Figure 4B). The C_max_ that appeared at the time point of withdrawal from injection was much lower than IV bolus administration (4.12 *vs.* 71.18 µg/mL), which was also well predicted (6.27 µg/mL).

**TABLE 4:** Predicted and observed pharmacokinetic AUC in rats, dogs, and monkeys receiving a single dose of ST-246.

Species	Dose (mg/kg)	Observed AUC (µg•h/mL)	Predicted AUC (µg•h/mL)	Ratio
Rat	2	3.00	2.99	0.997
	5	6.70	7.48	1.116
	20	30.39	29.12	0.958
Dog	1	1.91	1.90	1.000
	2.5	5.06	4.76	0.943
	10	16.51	19.02	1.158
Monkey	3	5.36	5.36	1.000

### 3.5 PBPK Model Validation in Dogs and Monkeys

The constructed rat PBPK model performance was validated by the available dog and monkey PK results. In the dog and monkey PBPK model, *CL* was obtained from the *in vivo* PK study, the volume of distribution was generated from transferred and un-transferred *K*_*p*_ values of rats. Predicted *V*_*ss*_ values for dogs and monkeys using transferred *K*_*p*_ values of rat were 3.44 and 1.295 L, respectively, while *K*_*p*_ values of rat led to *V*_*ss*_ of dogs and monkeys of 10.266 and 5.25 L, respectively, which was closer to observed *V*_*ss*_ values of 0.99–1.80 and 1.73–1.92 L/kg in individual PK studies. Therefore, transferred *K*_*p*_ values were not involved in this case. Under such conditions, both dog and monkey PBPK models well predicted the observed PK profiles *via* IV bolus and IV infusion (Figures 4C,D; Table 5). The good performance of this PBPK model in dogs and monkeys indicated that the basic assumption in animals is rational, and the extrapolation of the volume of distribution using *K*_*p*_ values of rats across species is feasible.

**TABLE 5:** Predicted and observed pharmacokinetic parameters in rats and monkeys receiving IV infusion of ST-246.

Species	IV-infusion	AUC (µg•h/mL)			C_max_ (µg/mL)		
	Dose (mg/kg)	Observed	Predicted	Ratio	Observed	Predicted	Ratio
Monkey	3	5.28	5.29	1.002	1.20	1.09	1.038
Rat	5	6.56	7.48	1.140	4.12	6.27	1.486

### 3.6 Human PBPK Simulation and Dose Regimen Prediction

**FIGURE 5:**
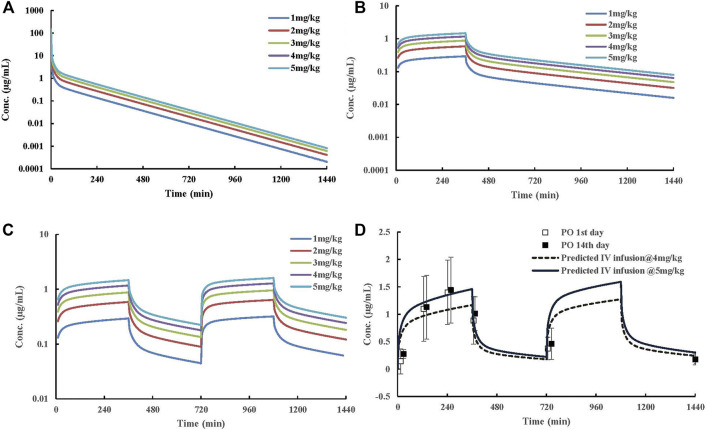
Predicted dose-dependent exposure of ST-246 in a virtual typical Chinese after IV, 6 h IV infusion QD, and 6 h IV infusion BID to capture the clinically relevant PK curves after oral dosing at 600 mg. **(A)** predicted human PK profiles post IV dosing; **(B)** predicted human PK profiles post 6-h IV infusion QD; **(C)** predicted human PK profiles post 6-h IV infusion BID; **(D)** predicted human PK profiles post 6-h IV infusion BID at 4 and 5 mg/kg dosages and observed clinically relevant oral PK profiles at therapeutic dose (600 mg). Clinical data were obtained from the literature. The solid square represents plasma concentration from the 1st day, and the hollow square represents plasma concentration from the 14th day.

Human PK projection of the ternary cyclodextrin complex of ST-246 from animal pharmacokinetics was finally conducted using this validated PBPK model. The simulations of ST-246 in humans *via* IV bolus, 6h-IV infusion QD, and BID over 1–5 mg/kg dose range are shown in Figure 5. As indicated in the “Materials and Methods” part, the clearance of humans was extrapolated from animal clearances, and the volume of distribution was generated from the integration of tissues with *K*_*p*_ values of rats (161.257 L). Proportional exposures (Table 6) were predicted based on the linear clearance and volume of distribution. The reported PK profiles of ST-246 after oral dosing at the therapeutic dose (600 mg) on the 1st day and the 14th day with mean C_max_ of 1.44 µg/mL and C_min_ of 0.779 µg/mL led to good efficacy and tolerance. The simulated PK behaviors of ST-246 demonstrated that 6-h IV infusion together with BID resulted in smoother PK curves. Obtained C_max_ and C_min_ were 1.27–0.178 µg/mL and 1.59–0.222 µg/mL for 4 mg/kg and 5 mg/kg, respectively. The comparison indicated 5 mg/kg IV infusion for 6 h and BID is an appropriate dosing regimen. The close T_max_ (6 h *vs.* 4 h) and C_max_ (1.59 µg/mL *vs.* 1.467 µg/mL, Chinsangaram et al., 2012) would produce reliable efficacy and avoid the occurrence of high concentration associated toxicity after IV bolus. Pop PBPK modeling further showed that 90% CI of C_max_ in plasma is within the range of 1.334–1.980 µg/mL, at dosage of 5 mg/kg following BID 6-h IV infusion.

**TABLE 6:** Predicted dose-dependent pharmacokinetic parameters of ST-246 in humans after IV, 6-h IV infusion QD, and 6-h IV infusion BID administration.

Administration pattern	Dosage (mg/kg)	1	2	3	4	5
IV QD	C_max_ (µg/mL)	36.26	72.53	108.8	145.1	181.3
	T_max_ (h)	0	0	0	0	0
	AUC (µg*h/mL)	2.41	4.83	7.24	9.65	12.07
IV infusion QD	C_max_ (µg/mL)	0.29	0.58	0.87	1.17	1.46
	T_max_ (h)	6	6	6	6	6
	AUC (µg*h/mL)	2.41	4.83	7.24	9.66	12.07
IV infusion BID	C_max_ (µg/mL)	0.32	0.63	0.95	1.27	1.59
	T_max_ (h)	6, 18	6, 18	6, 18	6, 18	6, 18
	AUC (µg*h/mL)	4.79	9.59	14.38	19.17	23.97

## 4 Discussion

Tpoxx (an immediate-release opaque hard gelatin capsule containing 200 mg of ST-246 plus excipients) by oral administration achieved FDA approval to treat pathogenic orthopoxvirus infections of humans in 2018 (Quenelle et al., 2007; Merchlinsky et al., 2019). Considering IV administration of ST-246 is an essential supplement for critical patients who have difficulty in swallowing oral formulations (Chen et al., 2011), a novel ternary cyclodextrin complex formulation is developed. Since different administration routes of the new formulation will lead to different PK profiles and clinical outcomes, preclinical pharmacokinetics were reevaluated comprehensively. In addition to IV bolus administration, IV infusion was also conducted to mimic the oral PK profile at therapeutic dosage. Finally, using the PBPK model, the pharmacokinetic behaviors of ST-246 post-IV dosing in experimental animals were extrapolated to human pharmacokinetics, to aid the decision of appropriate administration schedule for first-in-human trials.

PBPK is a mechanistic-based modeling approach based on mass-balance computation for organs and tissues. It is made up of compartments corresponding to different tissues in the body, connected by the circulating blood system (Basu et al., 2020). A successful PBPK model comprises three major components: system-specific properties, drug properties, and the structural model (Rowland et al., 2011). Commercial PBPK packages have made system-specific data available. The structure of the PBPK model is based on reasonable assumptions. Drug properties include physicochemical parameters and key pharmacokinetic parameters. Systemic clearance (*CL*) and volume of distribution (*V*_*ss*_) are critical PK parameters. *CL* describes how fast the drug would be eliminated from the blood; *V*_*ss*_ describes the relationship between drug concentration measured in plasma or blood to the amount of drug in the body at equilibrium (Murad et al., 2021). *CL* together with *V*_*ss*_, can be used to estimate elimination half-life and mean residence time, which define the concentration–time profile of a drug if it is administered *via* IV bolus.

Generally, extrapolated *CL* and *V*_*ss*_ using a bottom-up approach by *in vitro* to *in vivo* extrapolation (IVIVE) scaling are commonly accepted during PBPK model construction. For some extensively metabolized drugs, the liver clearance may be easily scaled from mechanistically well-stirred or parallel-tube models (Houston, 1994). ST-246 has been testified to be relatively stable, and its metabolism is negligible by any cytochrome P450 enzymes (Jordan et al., 2010). So, it is difficult to obtain *in vivo* clearance of ST-246 scaled from *in vitro* systems by an IVIVE-based bottom-up approach. During the current PBPK model building process, comprehensive preclinical pharmacokinetic studies were conducted and the results were fully utilized. The obtained systemic clearance of ST-246 in individual animal PK studies was directly put into the PBPK model, with kidney being the site of clearance.

On the other hand, although the solubility of ST-246 is low, it is highly permeable and no evidence has shown transporter involvement in the membrane diffusion (Jordan et al., 2010). Therefore, we used a perfusion-limited model that assumes passive membrane diffusion and homogeneous tissue concentration of ST-246 quickly achieved with blood flow (Jones et al., 2011). *V*_*ss*_ was extrapolated from measured *K*_*p*_ values of major tissues (brain, adipose, spleen, heart, lung, muscle, kidney, liver, and intestine) and fitted *K*_*p*_ values of other tissues (skin, marrow, reproductive organ, and rest of body) in rats. The extrapolated volume of distribution (0.101 L) was close to the observed value (0.0924 L) in the rat PBPK model. Meanwhile, we also attempted to apply methods by Poulin and Thei, Rodgers, and Lukacova to fit *V*_*ss*_ (Poulin and Theil, 2002; Rodgers and Rowland, 2007; Murad et al., 2021). Results showed that all the predicted *V*_*ss*_ values of ST-246 from the three methods dramatically overestimated the observed values. The measured *K*_*p*_ values of major tissues (Table 2) also indicated relatively limited tissue partition of ST-246. In our dog and monkey PBPK models, using *K*_*p*_ values from rats, extrapolated *V*_*ss*_ values (10.266 and 5.25 L, respectively), were close to *V*_*ss*_ observed from the intravenous PK study (11.04 ± 0.19 and 5.76 ± 1.41 L, respectively). Therefore, in the case of the current PBPK model of ST-246, consistency between observed and extrapolated *V*_ss_ across preclinical species suggested that tissue partition can be fixed, when the plasma protein binding rates are relatively high across the species. In addition, PK profiles of rats and monkeys *via* IV infusion were also well predicted in the current PBPK model. According to these results, the confidence in the human *V*_ss_ and clearance prediction and assumptions behind the model are warranted.

Human PK estimation for IV administration based on the human PBPK model is the first step of model-aided drug development of the new formulation of ST-246. Clearance was directly extrapolated from *in vivo* clearance of rats, dogs, and monkeys by allometric scaling. *V*_ss_ was acquired from transferred *K*_*p*_ values of 13 tissues. Allometric scaling of *V*_ss_ using observed *V*_ss_ of experimental animals was simultaneously performed. The extrapolated human *V*_ss_ was 158.5 L, which was very close to the predicted *V*_ss_ (161.257 L).

The prediction of human PK *via* IV bolus is the first step in the clinical translation of the novel ST-246 formulation. High concentration after IV dosing raises safety concerns, and subsequent rapid clearance could not benefit the maintenance of efficacy (Jordan et al., 2010). On the other hand, the adjustment of IV dosing regimen through the IV route is convenient and feasible. In order to identify the optimal IV administration regimen, we used the PK profile of ST-246 at therapeutic dosage (600 mg) after oral administration as the reference curve. Comparing the PK profiles simulated by the PBPK model at different administration regimes, we found that plasma curves obtained by extending the infusion time from 4- to 6-h matched the reference curve better. Ultimately, 6-h IV infusion at 5 mg/kg together with BID for a 70-kg adult yielded a PK curve most similar to the reference PK profile.

Artificial intelligence and data science have been broadly utilized across many fields, and biomedical research is never an exception (Theil et al., 2003; Yan et al., 2022). As a mathematical model, PBPK modeling has the capacity to extrapolate from known pharmacokinetics to unknown ones based on an algorithm that calculates *in vivo* physiological processes of a drug (He et al., 2019; Adiwidjaja et al., 2020; Singh et al., 2020; Kang et al., 2021; Kato et al., 2021). PBPK simulation has been playing an increasingly important role in drug R&D (Zhao et al., 2011; Zhuang and Lu, 2016; Li et al., 2020). A thorough understanding of the ADME processes of an individual drug is necessary for PBPK model construction (Li et al., 2021). In this case, the PBPK model structure was first built based on previous DMPK knowledge of ST-246. However, the commonly used bottom-up approach to obtain clearance and *V*_*ss*_ did not work. Both clearance and *V*_*ss*_ were generated by using the top-down method. Meanwhile, plasma protein binding (*f*_*up*_) and *R*_*b/p*_ in different species were included to transfer the *V*_*ss*_ across species. Model-aided drug development facilitates clinical translation. Although IVIVE scaling approach is a useful methodology to construct a mechanistic PBPK model, the top-down method integrated with species-specific extrapolation is still a good solution when IVIVE-linked clearance and volume of distribution are not available. Therefore, during PBPK modeling development, it is very important to apply available data while balancing scientific rigor and flexibly.

## 5 Conclusion

Taken together, to accelerate the development of ternary cyclodextrin complex of ST-246, a whole PBPK model was built and validated based on comprehensive preclinical studies and available knowledge to optimize the dose regimen of IV infusion for the treatment of severe smallpox. This mechanistic PBPK model well described the animal PK behaviors of ST-246 *via* intravenous injection and infusion. It appeared that 6h-IV infusion at 5 mg/kg BID produced similar C_max_ and AUC as oral administration at therapeutic dosage (600 mg) based on the translational human PBPK model. It will provide insights into the potential clinical application of a novel injectable ST-246.

## Data Availability

The raw data supporting the conclusion of this article will be made available by the authors, without undue reservation.
